# Obese persons’ physical activity experiences and motivations across weight changes: a qualitative exploratory study

**DOI:** 10.1186/s12889-015-2456-0

**Published:** 2015-11-14

**Authors:** Andrea E. Bombak

**Affiliations:** Cumming School of Medicine, Health Sciences Centre Foothills Campus, University of Calgary, 3330 Hospital Drive NW, Calgary, Alberta Canada

**Keywords:** Obesity, Physical activity, Stigma, Weight changes, Health-at-every-size

## Abstract

**Background:**

Obese individuals are encouraged to participate in physical activity. However, few qualitative studies have explored obese individuals’ motivations for and experiences with physical activity.

**Methods:**

The physical activity experiences of self-identified obese or formerly obese persons (*n* = 15) were explored through in-depth, semi-structured, audio-taped, repeated interviews and ethnography over one year. Participant observation occurred at multiple sites identified by participants as meaningful to them as obese persons. Data from interview transcripts and fieldnotes were analyzed via thematic content analysis.

**Results:**

Underlying goals for engaging in physical activity were diverse. Emergent motivation themes included: protection, pressure, and pleasure. Participants were protective of maintaining functional capacity, establishing fit identities, and achieving weight loss. Participants also discussed feelings of excessive pressure to continue progressing toward weight and fitness goals. Enjoyment in physical activity was often a by-product for all participants and could become a sought-after endpoint. Finding an environment in which participants felt safe, accepted, and encouraged to be active was extremely important for continual engagement.

**Conclusions:**

Obese individuals enjoyed physical activity and were concerned about maintaining functional fitness. Stigmatization and untenable goals and monitoring could disrupt physical activity.

## Background

Obesity is defined as having a body mass index (BMI) that exceeds 30 kg/m^2^. The etiology of obesity is often presented as produced by a simple imbalance in caloric intake versus expenditure. This depiction can lead to the value-laden presumption of larger individuals as necessarily over-indulgent and inactive [1], and this personal attribution can exacerbate already widespread stigma [2–5]. However, obesity need not preclude the development of an athletic identity or enjoyment in physical activity; cardiorespiratory fitness can be a more powerful predictor of mortality risk than BMI; and physical activity can reduce morbidity risks independent of weight loss [6–15]. Such findings have helped support and sustain the Health-at-Every-Size (HAES) movement that argues for a focus on promoting self-care behaviors for individuals of all sizes, such as enjoyable physical activity and healthful eating, rather than weight loss outcomes [6, 7]. Importantly, while the independent benefits of physical activity among obese individuals are well documented [16–21], little is known concerning what motivates and hinders activity in obese individuals, how fat corporeality may impact on activity, and how positive or negative experiences in activity contexts may influence these issues [22]. While many studies focus on activity with an explicit focus on weight loss outcomes, most weight loss is transitory [23], and obesity is increasingly recognized as a chronic state [24]. A greater understanding of obese persons’ motivations and experiences respecting physical activity is required, in order to produce messaging and interventions that are resonant with the target population. This study provides these insights among obese persons with diverse weight trajectories and perspectives on weight loss, rather than presenting obese persons as necessarily characterized by the same weight histories and goals. Data were collected over a year to generate insight into change, rather than treating obesity as a stagnant condition. Findings from this study may facilitate programs and messaging that appeals to, and meets the needs of, a broad spectrum of obese persons. The ethnography from which this study derived was designed with a focus on health and quality of life. The importance of physical activity, experiences with physical activity, and motivations underlying participation or non-engagement in physical activity spontaneously emerged in every interview and during participant observation. These data were subject to a focused secondary analysis for this paper. Therefore, the purpose of this paper is to describe obese and formerly obese persons’ experiences with physical activity and motivators of physical activity that emerged when discussing health and quality of life over one year.

## Methods

### Design

The article is derived from a larger one-year ethnographic study in a mid-size Canadian urban centre [25]. The study was approved by the University of Manitoba’s Health Research Ethics Board, and all participants gave informed consent prior to participating. The study design epitomizes the shift from traditional ethnography’s focus on a local, contained culture to a focus on critiquing cultural and temporal processes, discourse, subjectivities, and new forms of rationalities [26–28]. The study sought to investigate the lives of obese persons by capturing an emic perspective through long-term engagement in participants’ lives, in-depth interviews, and participant observation undertaken at sites identified as relevant by participants. Such an approach epitomizes Marcus’s “follow the people” technique of conducting multi-sited ethnography ([29]:106). By focusing on every day, mundane aspects of culture in persons’ lives, this approach is valuable for illuminating the power relations that influence obese individuals’ possibilities for wellness [28, 30]. The study’s qualitative longitudinal design and its use of repeated interviews allowed for a major focus on change over time, fluidity of the body, and contingency of bodily perspectives to emerge [31, 32].

### Research questions

During data collection, interview guides focused on addressing the following research questions:

1. How do participants view their own health?
2. How do participants view obesity and health?
3. How do participants’ views on healthy behaviors influence their everyday lives?
4. Do participants feel stigma? If so, how do they cope with said stigma?
5. How do participants’ views on health and obesity change over time and different weight trajectories?

The analysis on physical activity-related data specifically sought to address the following research questions:

1. What do obese persons prioritize with respect to physical activity?
2. What motivates obese persons to initiate, persist in, or discontinue physical activity?
3. Do obese persons’ experience stigma in physical activity contexts? If so, how do they cope with said stigma?
4. How do obese persons’ views on physical activity change over time and different weight trajectories?

### Participants

Recruitment occurred via online mailing lists and posters at multiple sites, following ethics approval from the researcher’s institutional Health Research Ethics Board. Study participants (*n* = 15) were self-identified obese and formerly obese adults, males (*n* = 2) and females (*n* = 13), 18 years of age or older. Maximum variation was sought on perspectives of obesity, health, and weight loss. Purposive sampling ensured that participants hoping for weight loss, fat acceptance advocates, and HAES proponents were all recruited by probing for attitudes during screening contacts and initial interviews. Recruitment proceeded until the sample included individuals holding all these views. Approximately 80 % of participants were Caucasian. However, a wide array of participants’ socioeconomic status, living conditions, and ages (range: 30s-60s) were included. Because of the hospital-based nature of one of the electronic mailing lists used in recruitment, many participants were affiliated with the broader medical community or had been prior to retirement or other alteration in employment status. A diverse range of occupations was still present even among those participants broadly affiliated with healthcare including: individuals who worked from home on medical records, accountants, administrators, home care aides, social workers, and providers. Participant characteristics are summarized in Table 1. However, to ensure participants’ confidentiality, detailed demographic data is not reported; the recruitment pool is small and some noticeable body transformations and narratives may reveal participant identities, if linkable to demographic details. Most important to the aims of the study, a wide variation was achieved in participants’ weight trajectories, perspectives on obesity, and health behaviors. Such categories are necessarily arbitrary, contingent, and fluctuating. However, at the start of the study, approximately two-thirds were hoping to lose weight or maintain a weight loss, while a third of participants were striving to achieve a state of body acceptance. Individuals described losing weight prior to the study or lost weight throughout data collection, however; only two individuals would be considered formerly obese. To ensure confidentiality all participants were assigned participant numbers and pseudonyms upon enrolment in the study. Those pseudonyms are used in this article.

**Table 1:** Participant Characteristics

Age (Range)	Sex (n)	Household	Ethnicity	Occupation	Weight goals
30s-60s	Female = 13	Partner = 8	Caucasian	HC = 6	WL = 7
		No Partner = 7	(*n* = 12)	HC–U = 2	ML = 3
	Male = 2	Has children = 8	Other (*n* = 3)	HC–P = 5	A = 5
				Other = 2	

*HC* Healthcare employee, *U* Unemployed, *P* Healthcare Peripheral

*WL* Weight loss, *ML* Maintain weight loss, *A* Accept current weight

### Interviews

All 15 participants were interviewed at least once. Subsequently, five participants were selected, based on achieving maximum variation in perspectives and experiences, for three repeated interviews (every three-four months) and participant observation. In total, 30 interviews were completed and analyzed for this study. Interviews lasted approximately 30 – 90 min. Interviews were conducted based on an interview guide, audio-taped, and transcribed verbatim. Interview guides were designed to address the research questions and to detect change over time in participants’ perspectives and behaviors. Repeated interview guides were constructed iteratively and individually tailored for particular participants. These guides included themes introduced from previous interviews and probes based on fieldnotes concerning particularly salient issues revealed by body language or verbal cues in previous interviews. Repeated interviews began with a form of member checking by clarifying issues from previous interviews and themes emergent in the iterative analysis, thereby enhancing interpretive and descriptive validity. This approach capitalized on the strength of qualitative longitudinal interviewing, which facilitates the study of change within a local, contingent context [31].

### Participant observation

Participants were asked about relevant sites at which to conduct participant observation. Detailed fieldnotes were recorded immediately following every interview and attendance at field sites. Fieldnotes were designed to maximize emic understandings, to be as contemporaneous as possible with data collection, and to focus on the interactional nature of the data collection process and the lived experiences observed [33]. Fieldnotes from participant observation were included in the overall data analysis to specifically address research questions. In all, participants were accompanied to fitness classes and in undertaking their fitness regimes; workplace and higher-end eateries; participants’ homes for post-exercise interviews and food and menu preparation; and grocery stores for weekly food procurement. Data organization was undertaken using the software package NVivo V. 10 (QSR International). Data were analyzed using a thematic content analysis approach incorporating both inductive and deductive procedures [34]. A coding list was generated following completion of the initial set of interviews informed by literature in the area, fieldnotes, and recurrent motifs evident in initial review of transcripts. Transcripts and fieldnotes were analyzed line-by-line and assigned to relevant codes. New codes were added as analysis proceeded. Codes were subsequently collapsed into categories, and categories were grouped into a series of themes. Themes were compared between participants and time points, and the effect of time and evidence of change was considered in all analytical procedures. Analysis was subjected to external auditing by members within, and external, to the author’s discipline, thereby contributing to the study’s theoretical validity. An audit trail was used to enhance interpretive validity and researcher reflexivity throughout analysis. This paper reports on themes that emerged when a sub-analysis following these procedures was conducted on physical activity-related data.

## Results

**Fig. 1:**
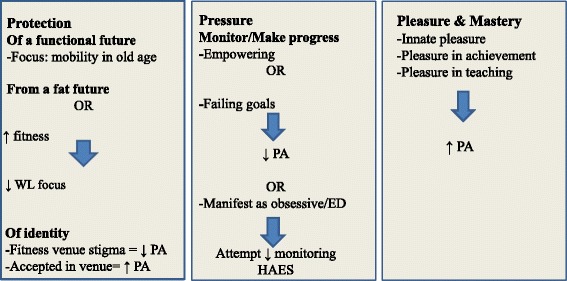
Inter-related motivators and inhibitors to obese persons’ physical activity. WL **=** weight loss; PA = physical activity; ED = disordered eating; HAES = Health-at-Every-Size; ↓ = decreased; ↑ = increased; = contributes to

Participants’ accounts of engaging in physical activity were multifaceted and complex. Most participants discussed numerous reasons for their often extensive engagement in physical activity. Participants expressed a desire to protect their bodies, engagement, and identities; pressure to adhere to standards or meet goals; and the pleasure derived from activity, involvement, or a sense of achievement. The major inter-related themes respecting physical activity motivators, inhibitors, and experiences described by participants are summarized in Fig. 1.

### Protection

#### Protection of a functional future

All participants described the importance of maintaining functional capacity. This was referenced repeatedly as a major component of health and a motivator of physical activity but was rarely a goal in itself. Rather, participants valued functioning because it facilitated social interactions, independence, or engagement in favorite activities:

*I want to be able to play with my son, I want to be able to chase my son, he gets so excited when you go chasing after him, or when he can chase after you.* (Pauline).

Participants’ worried that in the future their size might impinge on their ability to maintain engagement in favored activities, especially as they aged:

*I’d fallen while walking and had a serious concussion as a result. And I was only [middle-aged] at the time I think, and thought this is old lady business and this is not acceptable. Like I can’t even walk and not fall on my head*. (Amelia).

Worries that weight may be affecting their functional capacity could motivate weight loss attempts or more strenuous exercise, even amongst those identifying as more weight accepting. Among participants followed for a full year, most did sustain activity-prohibiting injuries. This may suggest that participants’ concerns over future immobility were not unwarranted. Or, efforts to forestall mobility problems, such as frequent rigorous activity, may have unintentionally contributed to inactivity-inducing injuries.

### Protection from a fat future

Other participants referenced the need to not gain weight in order to be thinner and/or healthy in the future. Participants hoping for weight loss viewed physical activity as a means of controlling weight through caloric expenditure. Todd, for example, had recently achieved a significant weight loss and attributed this explicitly to remaining hyper-vigilant respecting his caloric intake and exercise-induced caloric expenditures. Like all participants trying to maintain weight loss, he was very active. He considered rectifying lack of awareness of calories as key to public health and focused strongly on an energy balance model of obesity:

*I think the problem with society is they don’t know how much a calorie is, and if I was gym teacher . . . I would take all the kids to McDonalds and I would be like, “get anything you want…but you have to burn it off after”. So then you take these kids …and you literally you make them run laps until it’s gone … because society has no idea*.

Despite his willpower-centric approach to weight control, Todd also expressed frustration with how little he could eat and still maintain his weight and with his weight plateaus and fluctuations. Furthermore, he referenced pleasure he derived from physical activity, independent of weight loss, and he missed the social and physical aspects of physical activity when he experienced a debilitating injury during the data collection period.

The prioritization of weight loss was one of the most changeable aspects of participants’ physical activity motivations. For some more weight-accepting participants, incidental weight loss could promote re-engagement in weight loss efforts. However, some participants became more weight-accepting over time. As participants achieved higher levels of fitness and health, with or without weight loss, a decreased focus on weight could emerge. For example, Amelia experienced only very gradual moderate weight loss but notable improvements in fitness; she underwent what she termed ‘*a surrender*’ by the last interview. In describing a contributor to her heightened acceptance, she described her “gym experience” and her pursuit of physical activity aims:

*The people that I was working with there* [fitness staff at her physical activity venue] *were so health focused that that helped me to shift and learn and feel potential in being healthy at this weight without feeling like I had to change my weight.*

Participants were therefore highly concerned that their futures be characterized by freedom from immobility. For some participants, there was also an explicit focus on a thin future, but this emphasis could shift with accrual of other benefits from activity.

### Stigma and identity protection

Participants experienced a great deal of stigma, and their likelihood of physical activity was affected by their feelings of inclusion within particular fitness environments. Participants’ involvement in physical activity could have profound effects on their wellbeing. For example, Amelia had a negative experience with a wilderness activity group:

*I’d been on [another trip] maybe 15 years ago where the hiking club actually left me on a mountain because I couldn’t keep up, and [they] said you can sit here and wait for us to come down and get you, or you can go down by yourself. But you’re not coming with us; we’re not waiting for you anymore.*

Subsequent to an injury, Amelia joined a small private gym that had a major effect on her activity levels, perceived fitness, and social wellbeing. She described the degree of acceptance she felt as a larger, middle-aged woman in this locale. Her phone and home contained photos of herself and individuals she met at the gym. In participating with Amelia in one of her workouts, I was introduced to all the present employees and watched her greet a fellow middle-aged exerciser enthusiastically. She instructed me in her obviously familiar exercise regime and commented on liking her appearance in the gym mirrors.

Some participants did not find their physical venues as welcoming. Hannah, a former athlete enjoyed the exercise component of attending the gym but was weary of her visibility as a large woman:

*… It’s kind of intimidating … you’re doing something that’s making your body move in all sorts of ways that is not attractive, and you feel like everyone’s going to see this. So maybe if there were group classes… you’re in a group where you’re going to have support. And then if that group were people who are …of a certain weight like you… you don’t feel like you’re the only one*.

I accompanied Christine to her fitness centre. It was part of a chain and plastered with signs proclaiming a welcoming stance toward all bodies. Christine greeted her class instructor with a wide smile and wave, and the instructor described staff members’ fondness of Christine. Classmates encouraged our staying for a group fitness class and chatted with us after the class. Her feelings of approval and approbation within this context were apparent and reinforced in the interview following the interview. Despite this, Christine, who had a long-standing physical disability, expressed a wish that her fitness centre was more diverse in terms of ability status.

Overall, participants’ had varied experiences of inclusion or exclusion in physical activity contexts. Participants’ subjective interest and enjoyment in physical activity was often resilient. Participants often persisted in, or resumed activity despite negative experiences.

### Pressure

Some participants described a seemingly compulsive urge to achieve or to progress in levels of fitness. Hyper-vigilance concerning ‘tracking’ activity was mentioned by a number of participants with varying views on weight and health. Some participants focused on monitoring weight, others recorded progress in fitness, and others rigorously tracked food intake, sometimes in online fitness communities. Participants were especially dedicated to trying to following evidence-based weight loss advice. The dominant paradigm underlying most weight loss messaging is the need to rigorously monitor caloric intake and expenditure [1]. For some participants, such as Rachel, this monitoring seemed to be a component of her largely empowering and pleasurable ‘health journey’. For other participants, these actions were characterized by a sense of unwilling pressure. Indeed, some participants linked their past or present focus on monitoring weight, activity, and food to disordered eating. Melissa recounted periods of what she perceived to be problematic restrictive eating and over-exercising (up to two hours per day). She described her transition to what she considered a more “natural” and “healthy” approach of engaging in physical activity that appealed to her at the moment, rather than her prior more stringent activity routines:

*I just feel like going for a walk today, versus … I need … x amount of cardio and x amount of weight, so I better be at the gym and I better do this machine and that machine…*

Joanne, a former martial artist, found focusing on competition and progress anxiety-provoking. She felt this approach might be hampering her weight loss goals:

*I tend to be a … collector of awards in the past where I would aim for a goal and I would just keep on hammering and hammering away at it until I got something out of it. Like doing… so many miles and say if I had run two miles this week I wouldn’t allow myself to run less than two miles the following week.*

Some participants, over the whole course of data collection, tried to develop new approaches to hyper-vigilance. They tried to shift to less monitoring, if they felt it was hampering their overall wellbeing or weight loss goals. Many such participants explicitly aligned with the HAES movement, often encountering its tenets during disordered eating treatment.

Other participants, worried that ‘complacence’ was damaging their weight loss efforts, tried to resume hyper-vigilance. Many participants’ described this pressure as a motivator for the type and degree of their physical activity. For some, this was a welcome source of stimulus; for others, it was experienced as a health-damaging force.

Importantly, participants’ feelings of pressure respecting fitness and weight were affected by weight and fitness changes evident over the course of data collection. Participants’ goals shifted with body and lifestyle changes, particularly with respect to fitness and activity, and this could de-centre weight loss’s value. Participants who were active throughout data collection and experienced improvements in health and/or weight loss or stabilization, often expressed a de-emphasis on weight-centric goals. These participants’ prioritization of future health, irrespective of weight loss, remained unaltered.

### Pleasure and mastery

Participants described other rewards of physical activity, including enjoyment, socializing, and feelings of accomplishment. For some participants, these were pleasant by-products; for others, these rewards constituted the primary objectives for engagement. Christine described the enlivening effect of physical activity on herself and her daughter:

*My daughter . . . she can be… oh, just a terrible person. Just so down on herself and down on life. And we go to that pool on Wednesday; that Wednesday night’s going to be wonderful. And me too, I have more. ..even though I get tired [at the gym], I have more energy.*

Clarissa, an adamant advocate of HAES, exhibited another source of pleasure evident among participants, that of educating. I participated in a class taught by Clarissa. The confidence and enjoyment she expresses below was also evident when other participants taught me new activities:

*But the thing that allows me to enjoy it the most is that I’ve been teaching for several years and I’ve had students that have been with me for several years now and I’ve watched them come to a place of comfort in their bodies. And that’s amazing and just being able to accept and not put a limitation on themselves . . . the limitations are often there because of what … It’s something we heard when we were three years old . . . and that just continues to play in our head, but through practice and you know … A practice of letting go, we can let go of some of those old tapes, right . . . That is very satisfying to me*.

A sense of mastery was another enticement for engagement in physical activity. Maintaining functioning was the main aim of Rachel’s physical activity; however, she enjoyed reaching new heights of physical accomplishment:

*I mean I have more like fun health goals like, you know, eventually being able to do a dead lift of my weight or squats . . . of my weight . . . And … I plan to complete a mini triathlon, a sprint triathlon this summer. . And I'm just doing that for shits and giggles*.

Participants, therefore, were capable of enjoying physical activity, meeting fitness goals, and instilling skills in others. Still, participants’ enjoyment in activity was often in danger of being eclipsed by fear or achievement-based pressures. Pleasure could be overwhelmed by an anxiety-ridden or compulsive urge to meet particular standards or pre-emptively strike against a purportedly debilitating future.

## Discussion

This ethnographic study explored the physical activity experiences of self-identified obese and formerly obese persons. Participants engaged in physical activity to protect their future health, for the sake of pleasure, and because they felt pressured to pursue health and weight aims in a stigmatizing environment. These priorities shifted over time, weight fluctuations, and activity engagement, but they remained in some iteration as the primary activity-related foci of most participants.

Obese persons’ concerns with physical functioning have been found in other studies [35–37]. Older obese African-American women, much like some participants in the present study, were more concerned with maintaining functional independence via physical activity, rather than solely weight-based goals [35]. Low income, obese mothers in Michigan expressed related priorities. Similar to participants in the present study, these mothers described health behavior motivators including concerns regarding future health issues, physical limitations, and an inability to interact with children [36]. Post-bariatric surgery patients also highlighted that the benefits of physical activity included a higher level of functioning that facilitated familial interactions [38]. While awaiting bariatric surgery, however, patients discussed how they needed to lose weight in order to engage in further physical activity [37]. Given high rates of weight loss recidivism [23] and still limited capacity for bariatric surgery, this is unfortunate; physical activity may be protective of functional status, even in the presence of weight gain [21]. Greater health promotion efforts encouraging physical fitness’s benefits on functioning, even irrespective of weight changes, may align with obese individuals’ own concerns and have profoundly beneficial wellness benefits.

Participants experienced a significant amount of stigma, and this affected their fitness identities and pursuits. This is similar to other studies, in which obese individuals felt judged for their sizes in activity contexts and this could de-incentivize health behaviors [36, 37]. Derogatory treatment and histories of disordered eating left some participants unwilling to pursue weight loss *per se*. By enhancing their fitness, speaking to these pursuits, and highlighting the injustice of obesity stigma, participants were able to protect their identities and unsettle some of the stigma directed upon them. Furthermore by focusing on fitness over weight loss, participants could work to protect their future functional capacity, derive pleasure from activity, and adhere to perfomative pressures, without feeling hopeless concerning static, cycling, or escalating weight.

Participants described enjoyment in physical activity that was not necessarily contingent on weight loss, regardless of whether their fitness endeavors were ultimately intended to achieve this end. Engagement and achievement in exercise may serve as its own motivator for ongoing activity [39]. Among adolescents, for example, personal fulfillment was the only independent motivator of activity, and, in fact, weight-based motivation was negatively associated with physical activity [40]. The pleasure-based activity motivations of participants in the present study strengthens the appeal for a greater focus on affect in body and physical activity related research [41, 42] and echoes findings from other studies on activity among obese persons [37, 38]. For some previously inactive participants in the present study, whole social relations, identities, and communities could emerge from physical activity.

The evident pleasure in activity demonstrated by participants reinforces the findings of other authors that athletic identities and material fatness are not mutually exclusive. Provided the opportunity, activity can be mastered and pleasurable regardless of size and despite previous stigmatization, as long as individual preferences concerning activity are taken into account, activities are appropriate, and environments are inviting [10, 12–15, 37, 43]. One aspect of making activity venues safe for obese participants frequently reiterated by participants in this, and other studies, is increasing bodily diversity in terms of gender, size, age, and ability [17, 37, 38, 44].

For some participants, pleasure related to physical activity was directly tied to making and recording progress. Heyes discusses the potentially enabling factors that may accrue from dieting in a commercial weight loss group [45]. The group offered a socially-approved and supportive venue in which women could be preoccupied with better knowing and enhancing themselves, even while trying to ‘correct’ non-normative bodies [45]. Many participants in the present study referenced the value of goal setting, rewards, and social supports as sources of motivation for activity.

Some participants expressed concerns that a focus on self-monitoring was dangerous to their overall wellbeing, based on prior disordered eating experiences. These participants struggled to balance pressures to be active, the pleasure they derived from activity and measuring their progress, and the worry this would lead to pathological behaviors. Similarly, patients waiting for bariatric surgery similarly asserted an” all or nothing approach” to physical activity and a need to meet potentially unrealistic standards of social contacts [37]. Overwhelming expectations and goals may ultimately discourage activity among obese persons. When weight goals are not met for overweight or obese women, despite beliefs in other benefits of physical activity, this can de-motivate exercise behaviors [17].

Overall, participants’ experiences of activity-derived pleasure were multidimensional, varied, and social. Innate experiences of enjoyment were always in danger of being overshadowed by the fear that their efforts were insufficient to achieve ‘wellbeing’ ([46]:23). This could lead to altered, ceased, or disordered activity patterns. That some participants were able to focus on overarching fitness and health goals, irrespective of weight fluctuations and plateaus suggests they are well-positioned to achieve the independent benefits of physical activity and fitness [16–21].

This study was characterized by strengths and limitations. The long-term engagement, participant observation, and repeated interviews helped enhance the trustworthiness of findings and their theoretical validity. Repeated interviews helped enhance rapport, produced more and richer data, allowed for organic member checking, and facilitated researcher and participant reflexivity while reflecting on past interviews. Furthermore, this research design provides greater focus on change than qualitative studies that only include one interview and therefore can present only a snapshot view of necessarily mutable bodily and attitudinal phenomena. Findings such as participants’ increasing sense of mastery, greater body acceptance, and shifting approaches to caloric vigilance were only apparent in the long term and highlight the advantages of using repeated interviews. During the larger study from which this data was derived, analysis was iterative and ongoing during repeated interviews. This allowed for member checking and clarification of issues from previous interviews during data collection, increasing descriptive and interpretive validity. Analysis was subject to ongoing external auditing, enhancing theoretical validity. Variability was sought in weight loss, health, and obesity attitudes. However, the sample was mostly Caucasian, middle-aged, presently obese women, many of whom were affiliated with the healthcare system. This is a major limitation of the study. Given the small and homogenous sample, the findings cannot be generalized. However, the findings largely align with studies conducted in other samples (reviewed above). Furthermore, the themes identified can be investigated in other samples to test their transferability, particularly as more research is needed exploring physical activity experiences and motivations among obese persons of different ages, ability statuses, races, sexualities, genders, ethnicities, and weight trajectories.

## Conclusions

In an ethnographic study on obese persons’ experiences and health perceptions, most participants were active. The study was novel in its inclusion of participant observation and focus on change over time, and inclusion of individuals characterized by diverse weight attitudes and trajectories. To the existing literature, this study provides insights into what motivates obese and formerly obese persons to be physically active and how relevant experiences shape their perception of physical activity and its relationship to health, quality of life, and obesity. Findings from this study may help inform physical activity programs and messaging for obese persons. In addition to weight loss, motivators for physical activity included maintenance of functioning and mobility; feelings of weight-based stigma and pressure to be active; and pleasure derived from activity. Finding an environment in which participants felt safe and encouraged to be active was extremely important, and instances of such inclusion had major ramifications on social wellbeing. Ultimately, most participants invoked all of protective, pressure, and pleasure-based motives for physical activity involvement. Some participants cycled through these motivations depending on material changes in their bodies. While participants had pleasure-related reasons for engaging in physical activity, this enjoyment could be overshadowed by the grip of caloric fear or dread of a demobilized future; a need for performative achievement or a thinner body; or the need to be a conspicuous exception to potentially stigmatizing anti-obesity messaging. Focusing on their health and fitness achievements and behaviors may have helped participants cope with their sizes in a stigmatizing climate. However, participants could still be overwhelmed by pressures to always be healthier or more active, and many participants had histories of disordered eating and activity practices.

These findings imply that to encourage physical activity among individuals with diverse weight histories and attitudes, physical activity messaging and programming may benefit from moving beyond a weight loss focus. Many participants in the present study found physical activity rewarding in itself. Disappointment in weight loss aims could be a disincentive to physical activity. Furthermore, physical activity messaging should feature a variety of bodies and abilities, as should activity venues and classes. Discrimination in such settings should be strictly disallowed, and pacing and goal-setting should be moderate and at the participant’s discretion. An inclusive model of physical activity for people of all sizes would focus on enjoyment, pleasurable accomplishment, and social belonging emergent during physical activity, rather than focusing on possibly unachievable or ultimately exclusionary endpoints. This study also highlighted areas requiring greater research. Few men participated in the present study and similar research; therefore, more in-depth, long-term research is needed in exploring men’s experiences of physical activity while obese. The experiences of physical activity following significant weight loss or gain also must be explored.

## Ethics, consent and permissions

Study was approved by the University of Manitoba Health Research Ethics Board.
